# Supplementary material for: Size Effect of a Piezoelectric Material as a Separator Coating Layer for Suppressing Dendritic Li Growth in Li Metal Batteries
Source: Nanomaterials (Basel). 2022 Dec 24;13(1):90. doi: 10.3390/nano13010090 (PMC9823885; doi:10.3390/nano13010090)
Supplement: Supplementary file 1 [file nanomaterials-13-00090-s001.zip › nanomaterials-2105177-supplementary.pdf]

nanomaterials

Supplementary Materials

Size Effect of a Piezoelectric Material as a Separator Coating Layer for Suppressing Dendritic Li Growth in Li Metal Batteries

Junghwan Kim [1,2,†], Kihwan Kwon [1,2,†], Kwanghyun Kim [1,2,†], Seungmin Han [1], Patrick Joohyun Kim [2,*] and Junghyun Choi [1,*]

1 Energy Storage Materials Center, Korea Institute of Ceramic Engineering and Technology, Jinju 52851, Republic of Korea
2 Department of Applied Chemistry, Kyungpook National University, Daegu 41566, Republic of Korea
* Correspondence: pjkim@knu.ac.kr (P.J.K.); jchoi@kicet.re.kr (J.C.)
† These authors contributed equally to this work.

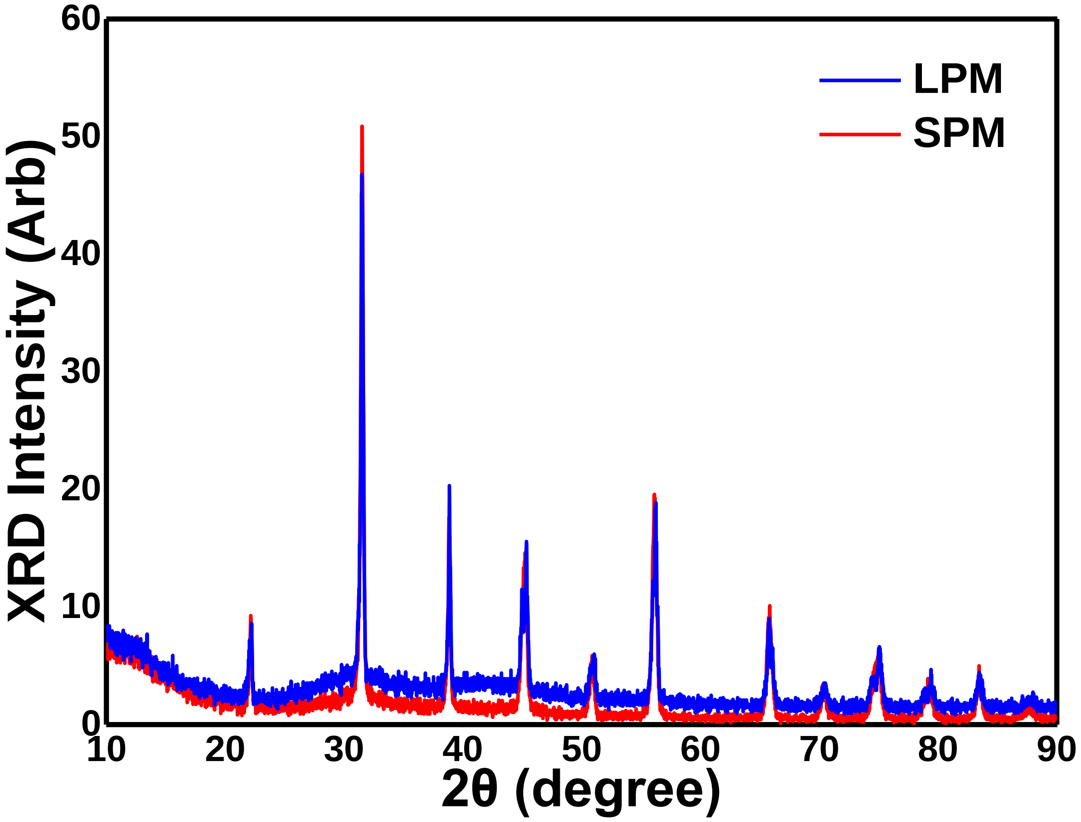

Figure S1. X-ray diffraction (XRD) of LPM and SPM.
